# Community health extension program of Ethiopia, 2003–2018: successes and challenges toward universal coverage for primary healthcare services

**DOI:** 10.1186/s12992-019-0470-1

**Published:** 2019-03-26

**Authors:** Yibeltal Assefa, Yalemzewod Assefa Gelaw, Peter S. Hill, Belaynew Wassie Taye, Wim Van Damme

**Affiliations:** 1 School of Public Health, the University of Queensland, Brisbane, Australia; 2 Department of Public Health, Institute of Tropical Medicine, Antwerp, Belgium

**Keywords:** Community health program, Health extension program, Primary health care, Universal health coverage, Ethiopia

## Abstract

**Background:**

Ethiopia has been implementing a community health extension program (HEP) since 2003. We aimed to assess the successes and challenges of the HEP over time, and develop a framework that may assist the implementation of the program toward universal primary healthcare services.

**Methods:**

We conducted a systematic review and synthesis of the literature on the HEP in Ethiopia between 2003 and 2018. Literature search was accomplished in PubMed, Embase and Google scholar databases. Literature search strategies were developed using medical subject headings (MeSH) and text words related to the aim of the review. We used a three-stage screening process to select the publications. Data extraction was conducted by three reviewers using pre-prepared data extraction form. We conducted an interpretive (not aggregative) synthesis of studies.

**Findings:**

The HEP enabled Ethiopia to achieve significant improvements in maternal and child health, communicable diseases, hygiene and sanitation, knowledge and health care seeking. The HEP has been a learning organization that adapts itself to community demands. The program is also dynamic enough to shift tasks between health centers and community. The community has been a key player in the successful implementation of the HEP. In spite of these successes, the program is currently facing challenges that remain to be addressed. These challenges are related to productivity and efficiency of health extension workers (HEWs); working and living conditions of HEWs; capacity of health posts; and, social determinants of health. These require a systemic approach that involves the wider health system, community, and sectors responsible for social determinants of health. We developed a framework that may assist in the implementation of the HEP.

**Conclusion:**

The HEP has enabled Ethiopia to achieve significant improvements. However, several challenges remain to be addressed. The framework can be utilized to improve community health programs toward universal coverage for primary healthcare services.

## Background

The Alma-Ata Declaration of 1978 identified primary health care (PHC) as the key approach to the attainment of the goal of “Health for All”. The PHC approach tackles the main health problems in the community through the provision of essential health services. The implementation of the PHC approach relies on health workers, including community health workers (CHWs). Community health worker embraces a variety of community health aides. They are members of a community who are chosen by community members or organizations to provide basic health and medical care to their community. As such, CHWs represent an important health resource with a potential to provide a reasonable level of health care [1].

Several countries have been implementing community health programs (CHPs), which provide basic health and medical care close to community, to increase access to and coverage of essential health services [2]. Ethiopia has been implementing a nation-wide CHP called health extension program (HEP) since 2003 [3]. Implementation of these CHPs, although they may have their own peculiarities, faces similar enablers and barriers for their effective implementation [4]. It is relevant that lessons from large-scale programs are synthesised to provide evidence of what works, what does not work and how can we improve performance. Currently, there is lack of evidence on the successes and challenges of the implementation of large-scale CHW programs which are included and implemented as an integral part of the national health system. It is therefore imperative to fill this knowledge gap toward improved and sustained implementation of large-scale and long-term CHWs program [4].

In a previous study in Ethiopia, we found that there were significant improvements in health outcomes during the era of the Millennium Development Goals (MDGs). There was a 67% reduction in under-five mortality; a 71% decline in maternal mortality ratio; a 90% decline in new HIV infections; a decrease in malaria-related deaths by 73%; and a more than 50% decline in mortality due to TB between 1990 and 2015 [5]. These successes in health outcomes were due to implementation of a mix of comprehensive strategies including improvements in health systems and overall socio-economic status in the country. The same study indicated that the HEP contributed to Ethiopia’s success in achieving the health MDGs [5]. However, the contribution of the HEP was not evaluated systematically. Hence, a number of questions remained to be answered by a systematic review and analysis of the existing evidence. The main questions that the program needs answer for include: What are the successes and challenges of the HEP between 2003 and 2018? What initiatives have been implemented to address the challenges and improve access to primary healthcare services?

The objective of this study is to identify successes and challenges in the implementation of the HEP in Ethiopia, find out initiatives undertaken to improve program challenges, and develop a framework, based on the review and synthesis, which may assist the country in strengthening its community HEP toward universal coverage for primary healthcare services.

## Methods

### Community health extension program of Ethiopia

**Fig. 1:**
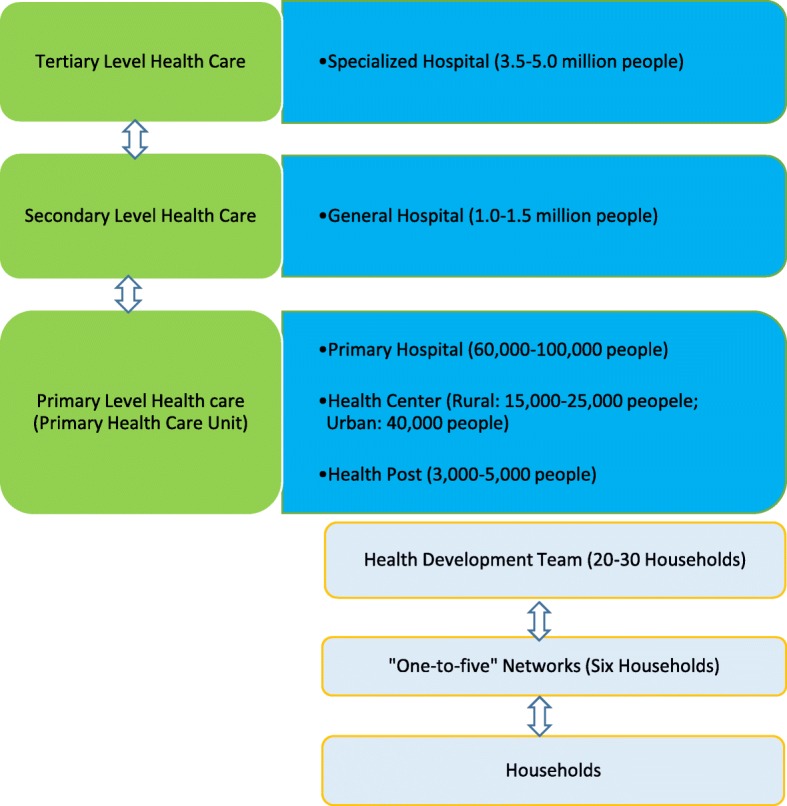
Ethiopian health care delivery tier

Ethiopia’s health service delivery is structured into a three-tier system: primary, secondary and tertiary levels of care. The HEP is positioned, implemented and managed under the umbrella of the primary health care unit (PHCU) (Fig. 1) [3, 6]. Two HEWs are assigned to one health post to serve a population ranging from 3000 to 5000 in a village (kebele). Five health posts and a health center work in collaboration and form the PHCU that serves 25,000 people. The health center serves as a referral center. The health post is under the supervision of the district health office and the kebele administration and receives technical support from the nearby health center.

**Fig. 2:**
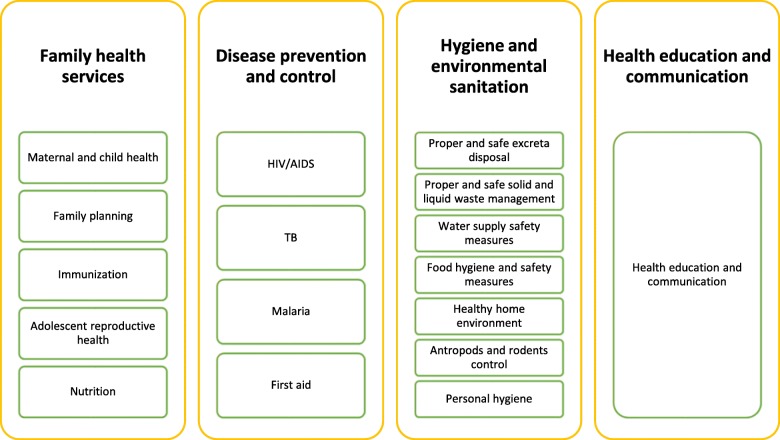
Packages of the rural health extension program of Ethiopia

The HEP was developed in a context where health outcomes and coverage of essential services were very poor, including low coverage of maternal and child health services. There was also a large disparity between rural and urban populations, and among demographic and socio-economic groups. Moreover, there was a critical shortage of skilled health workers, and weak coordination of service delivery [3, 6]. The program was launched at scale in 2003 with 17 packages under four areas (Fig. 2) in agrarian (rural) regions whose subsistence mainly depends on agriculture. The program was later adapted for pastoralist and urban regions in 2010 [3].

Upon completion of the 12-month training, HEWs are assigned as salaried government employees to health posts and work directly with households. HEWs map households and prioritize health problems of the village, and draft a plan of action. More than 42,000 government-salaried female HEWs are deployed in the country. HEWs provide key health services through static and outreach activities. They are expected to spend 25% of their working time conducting home visits and outreach activities and the remaining 25% at health post providing basic curative, promotive and preventive services [3, 7, 8].

The HEP has evolved over time; its implementation is currently supported by key actors: HEWs, model households (MHHs) (also called model families), the health development army (HAD), the community, and the government. Model households are those households that are trained in HEP packages, implementing these packages after the training, and able to influence their neighbours to adopt the same practices [8].

The HDA represents a systematic, organized, and collaborative movement through active participatory learning and actions to improve health. The HDA has been providing an effective platform to engage the community in the planning, implementation, monitoring, and evaluation of health and other programs in the country since 2012. A functional HDA requires health development teams (HDTs) that comprise up to 30 households residing in the same neighbourhood. The HDT is further divided into smaller groups of six members, commonly referred to as one-to-five networks. HEWs and Kebele administrations facilitate the formation of HDTs and one-to-five networks. Volunteer CHWs (including traditional birth attendants, health promoters, and reproductive health agents) help HEWs in mobilizing the community [8].

### Study design

We conducted a systematic review and synthesis of the literature, exclusively using peer-reviewed publications, from primary researches on the HEP in Ethiopia between 2003 and 2018**.**

### Literature search and selection

Literature search was accomplished in PubMed, Embase and Google scholar databases. Literature search strategies were developed using medical subject headings (MeSH) and text words related to the aim of the review: (Health extension worker OR Health extension program OR community health workers OR health development army OR health extension package) AND (performance OR success OR efficiency OR cost effectiveness OR challenge OR limitation OR gaps OR weakness OR attitude OR Failure OR cost OR strength OR monitoring OR evaluation) AND Ethiopia.

We used a three-stage screening process to select the publications. Two reviewers (YA and YAG) excluded articles based on their titles (stage one), abstracts (stage two) and assessed full texts (stage three) independently and in duplicate. Data extraction was conducted by three reviewers (YA, YAG and BWT) independently and in duplicate from all included sources using pre-prepared data extraction form. Disagreements at any stage of the selection process was resolved through discussion by the reviewers, with the involvement of a third reviewer if needed.

### Data analysis

We conducted an interpretive (not aggregative) thematic synthesis of studies. The analysis included three steps: the coding of text ‘line-by-line’; the development of ‘descriptive themes’; and the generation of ‘analytical themes’ [9]. The analysis was conducted to identify and synthesise successes, challenges, and any changes in program implementation over time. It enabled us to develop a framework which could be tested by further studies.

Meta-analysis was not conducted, as there was scarcity of studies on similar components of the HEP. We assessed the quality of the included studies in the context of the purpose of our review, and not in the context of the primary studies themselves, and emphasised the ability of the studies to answer our review questions.

## Results

**Fig. 3:**
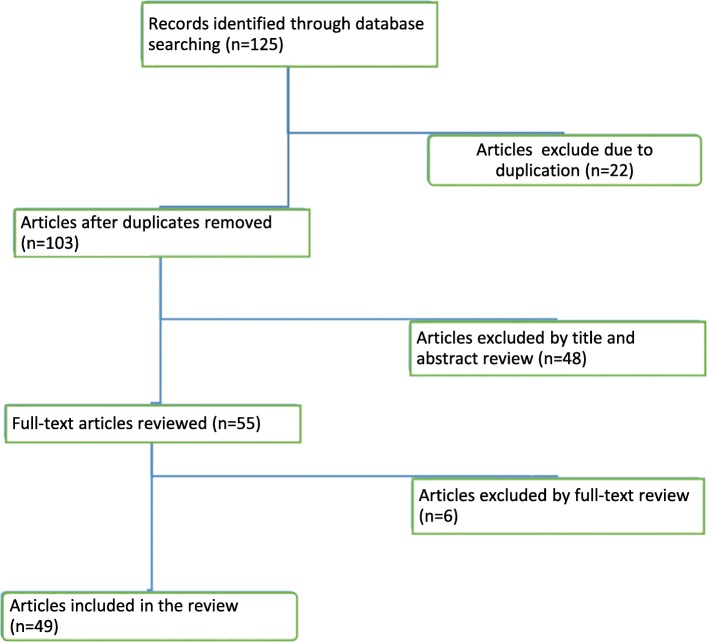
Flow diagram of literature search and selection strategy

The literature search initially identified 124 peer-reviewed papers published between 2003 and 2018. Twenty-two of these papers were found to be duplicates. Forty-eight papers were excluded by reading their titles and abstracts, as they were not related to the objectives of the study. Full-text review was then conducted on 55 papers; and, this excluded six papers as they were not directly related to the objectives of the study. Finally, this review included 49 papers published between 2007 and 2018 (Fig. 3).

The findings of the review and synthesis are organized and presented according to the objectives of the review: successes and challenges of the HEP over time; interventions to improve the implementation of the HEP; gaps that remain to be addressed by the HEP; and, framework to improve the implementation of the HEP toward universal coverage of primary healthcare services.

### Successes of the health extension program

A number of successes of the HEP were identified. The program had already achieved successes (in family planning, immunization, ANC, malaria, TB, HIV, and community satisfaction) in the first five years of implementation. There has been increased service utilization; improved knowledge and care seeking; increased latrine construction and utilization; enhanced reporting of disease outbreaks; and, high level of community satisfaction [10].

Completion of the health extension packages had an effect on health seeking behaviour of mothers for common childhood illness [11]. The program improved family planning, immunization, and ANC visits [12, 13]. Mothers from model households (MHHs) were 3.97 (95% CI, 3.01–5.23) times more likely to use contraceptives compared with mothers from non-MHHs [14]. HEWs were the primary source of information on long-active contraceptive methods in 71.8% (95% CI 67.0, 76.3) of the population [15].

Women who had been working towards graduation or graduated as model family were 2.13 times more likely to demonstrate good utilization of maternal health services [16]. For every unit increase in the program intensity score, the odds of receiving maternal and neonatal services increased: ANC by 1.13 times (95% CI 1.03, 1.23); birth preparedness by 1.31 times (95% CI 1.19, 1.44); receiving postnatal care by 1.60 times (95% CI 1.34, 1.91); and, initiating breastfeeding immediately after birth by 1.10 times (95% CI 1.02, 1.20) [17]. ANC attendance, at least four times, was significantly associated with visit by HEWs [OR = 3.46 (95% CI 3.07, 3.91)]. Women visited by HEWs during pregnancy were highly likely to attend postnatal care during the first three days [OR = 3.68 (95% CI 2.05, 6.59)] [18].

The proportions of children and women using insecticide-treated net (ITN) for malaria protection were significantly larger in HEP villages than in non-HEP villages. ITN utilization was 44% amongst women in HEP villages, while it was 8% lower without the HEP [13]. In general, there was high awareness regarding symptoms and use of preventive measures for malaria [19].

The involvement of HEWs in sputum collection and treatment improved smear-positive TB case detection (122.2% vs 69.4%, *p* < 0.001) and treatment success rate (89.3% vs 83.1%, *p* = 0.012 in intervention compared to control kebeles (villages) [20]. It was also found that people from MHHs [OR = 21.2 (95% CI 9.5, 47.3)], and home visits by HEW [OR = 2.8 (95% CI 1.2–9.6)] were significantly associated with referral for TB services [21].

The HEP also improved utilization of HIV testing [16]. Visit to HEWs and participation in community conversation affect utilization of voluntary counselling and HIV testing (VCT). The odds of VCT uptake among women who proactively visit HEWs were 1.40 (95% CI 1.13–1.73) times higher than among those women who did not visit HEWs. The odds of VCT uptake among women from MHHs were 1.50 (95% CI 1.03, 2.18) times higher than among those from non-MHHs [22]. There was a very high (92.7%) overall knowledge about HIV/AIDS, and HEWs were the main (73.3%) source of information [19].

The HEP had larger and significant impact on regular usage of latrines [13]. Latrine adopters were significantly more likely to report being advised by a HEW (*P* < 0.0001). Respondents who were advised by a HEW were more likely to have built a latrine [OR = 17.2 (95% CI 8.9, 33.0)] [23]. A two-week diarrhoea prevalence in two weeks in under-five children among model and non-MHHs were 6.4 and 25.5%, respectively. Proper latrine utilization and children’s stool disposal were more practiced by MHHs [265 (100%) and 247 (93.2%)] than non-MHHs [429 (83.1%) and 307 (58.1%)], respectively. Children from non-MHHs were 4.50 times more likely to have diarrhoea than children from MHHs [OR = 4.50 (95% CI 2.52, 8.03)] [24]. HEP facilitated community-led total sanitation (CLTS) in Ethiopia. HEW-facilitated CLTS was initially more effective than teacher-facilitated CLTS [25].

Assessment of community perspectives on HEP indicated that there were very few complaints by the community, except for curative care and delivery services [26]. There was positive feeling about the service offered by HEWs and they were preferable to other CHWs [27]. In one study, high satisfaction (mean 83.0 ± 18.2) and favourable interpersonal relationship (75.5%) were reported [28]. In another study, it was identified that HEWs had good relationships with the community. The mean score of overall community satisfaction with the urban HEP was 72.82(±22.09). The majority (67.4%) of respondents were satisfied with the services provided by the urban HEP [29].

### Challenges of the health extension program

In spite of these successes, the HEP had also challenges since the launch of the program. There were resource gaps, including medical equipment and drugs; limited supportive supervision; absence of a well-established referral system; high turnover of HEWs; absence of clear career structure for HEWs; unattractive salary scale; and, inadequate delivery and curative services in 2008 [10]. Some of these remain challenges to be addressed by the program.

In 2012, health posts did not have basic infrastructures like water supply, electricity, and waiting rooms for women in labor [12]. Data from service availability and readiness assessment, in 2016, also indicated that the mean availability of trace items for basic amenities, infection prevention, malaria diagnosis, and essential medicines at health posts was 37, 29, 52 and 46.5%, respectively. The overall general services readiness index at health post level was 46% [30].

Living and working conditions of HEWs were not conducive during the early phase of the implementation of the HEP, according to a study published in 2007 [31]. HEWs were deployed in remote areas where housing was very important in motivating and retaining them in the communities. The relationships between HEWs and other CHWs was not clearly established by 2008 [26]. A recent study, published in 2017, on job-related wellbeing indicated that stress and burnout were recognized among healthcare workers. Notwithstanding these, there were an unmet need for interventions to manage burnout or emotional difficulties [32].

In a study published in 2008, it was identified that the majority (88%) of HEWs had poor knowledge on danger symptoms and signs, and complications in pregnancy. Moreover, most HEWs did not feel confident enough to undertake delivery independently because of limited practice during the training [26]. Even later, in a study published in 2013, it was found that the skill and competency of HEWs to handle maternal health services was less trusted [28]. In another paper in 2015, only 50.5% of study participants perceived that HEWs were competent to deliver curative and delivery services [29]. A recent study identified that pre-service education did not prepare HEWs for all the tasks that comprise their scope of practice [33].

Studies, in the early phase of the HEP, indicated that the program reduced neither the incidence nor the medical care seeking behaviour to treat diarrhoea and cough for children. The studies also showed that the HEP did not have a statistically significant effect on delivery and postnatal care services in 2009 [13]. Utilization of clean and safe delivery was very low (19%) with only 1% of study participants giving birth at health posts in 2012 and 2013 [16, 34]. HEP was not associated with skilled deliveries, nor with some newborn health care indicators in 2012 [17]. Health facility delivery was not significantly associated with visit by HEWs during pregnancy [OR = 0.87 (95% CI 0.25, 2.96)] in 2013 [18].

### Initiatives to improve the health extension program

In order to address the above challenges of the HEP, the country implemented a series of initiatives, including the integrated Community Case Management (iCCM), Maternal and Newborn Health in Ethiopia Partnership (MaNHEP), health development army (HDA), and related trainings and mobile technologies. The integrated Community Case Management (iCCM) strategy increased care seeking for children with ARI, diarrhoea or fever. Care seeking increased from 19% at baseline to 38% at follow-up. Higher intensity of the HEP and other accessibility factors were associated with higher care seeking for childhood illnesses from HEP. Kebeles with higher density of HEWs were associated with higher utilization of iCCM services in 2014 [35]. Performance review and clinical mentoring meetings improved performance of HEWs [36]. Strong government commitment and leadership, and national coordination among development partners helped successful national iCCM introduction and scale-up [37]. A Cost-effectiveness analysis of Community-based Interventions for Newborns in Ethiopia (COMBINE) identified that management of probably severe bacterial infection in newborns by HEWs and CHWs at community level was estimated to reduce neonatal mortality by 17% and had cost savings [38].

Maternal and Newborn Health in Ethiopia Partnership (MaNHEP) improved maternal and newborn health care delivery. It was associated with improved coverage and completeness of maternal and child care and perinatal survival in 2014 [39]. Women who participated in two or more meetings had more complete care than women who participated in fewer than two meetings (89% vs 76% of care elements; *P* < 0.001) [40]. Mothers who had frequent household visits by HEWs were more likely to visit the health posts [AOR = 1.29 (95% CI 1.03, 1.83)] than mothers who did not get frequent visits. Mothers from MHHs were more likely to visit health post [AOR = 2.15 (95% CI 1.06, 4.37)] than mothers from non-MHHs [41]. Improvements in illness recognition and care seeking during MaNHEP were associated with factors such as family meetings, health facility birth, birth with a skilled provider, or HEW [42].

Communities with one health development army (HDA) team leader for at least every 40 households were associated with 12.4, 10.0, 8.4 and 7.9 percentage-points higher (*p* < 0.05) coverage of ANC, institutional deliveries, clean cord care and thermal care than those in communities with one HDA team leader for every 60 or more households in 2017 [43]. Identification of pregnant women through Women’s Development Groups (WDGs), and referral by ambulance to health facilities were facilitators to skilled birth attendance. With the support of WDGs, HEWs have increased the rate of skilled birth attendance by calling ambulances to transfer women to health centres in 2016 [44]. Provision of services that are culturally acceptable for women at health centers also facilitated health facility delivery [45]. There was a statistically significant upward trend for delivery and PNC in all facilities (*p* < 0.01) in 2015 [46].

Training to strengthen maternal and newborn health improved performance of HEWs. Post-training performance scores were significantly higher than immediate pre-training scores (*p* < 0.001) [47]. There was an overall strong retention of knowledge and skills among HEWs [48]. In 2017, over half of HEWs had learned about tasks that comprise their scope of practice through in-service training. HEWs perceived themselves as sufficiently competent to perform most tasks, with the exception of deliveries and management of supplies and stocks [33]. Mobile technologies are also assiting HEWs in the delivery of maternal health care services [49].

Initiatives were identified to improve mutual respect and collaboration between CHWs, HEWs and skilled birth attendants to help ensure timely consultation, referral and reduce delay [50]. A study on factors shaping interactions among CHWs in rural Ethiopia identified a core set of factors, including trust in co-workers, gender, and cadre, which were influential for teamwork across CHWs [51]. Trust and past training together are important relational factors for work interactions among diverse CHWs [52]. MaNHEP was associated with more and better interactions and relationships among HEWs, CHWs and the community. There were significant improvements in awareness, trust, and completeness of care [39]. The HDA supported HEWs in liaising with community members. Top-down supervision and provision of training improved relationships between HEWs and woreda health office in 2015 [53].

In spite of these initiatives and improvements in the HEP, there are still gaps that the program needs to systematically address. These gaps, which remain to be addressed during the Sustainable Development Goals (SDGs) era, are related to efficiency of the HEWs, performance of the HEP and social determinants of health.

### Time allocation and task analysis of health extension workers

HEWs have progressively become static that they spent less time in the community over time. In a study in 2008, HEWs indicated that 75% of their time was spent on health education and environmental health at community level [26]. Later, HEWs spent more than 70% of their time by making home-to-home visits. They provided family health services at the health post level (30%) [10]. In a study in 2014, it was found that HEWs divided their time between the health post (51%) and the community (37%), with 12% of their time spent elsewhere. In a study in 2017, out of the total time spent in providing health education and services, HEWs spent 43.8% of this time at health posts, 36.5% in community and households, 7.8% at schools or other outreach sites, and 12.9% of this time did not have a recorded site [54].

In a study in 2014, curative health activities represented 16% of HEWs’ time and health promotion and prevention represented 43% of their time. The remaining time (41%) was spent on travel, training and supervision, administration, and community meetings [55]. In a study in 2017, out of the total observed work time, HEWs spent their time providing health education or services (12.8%); participating in meetings and giving trainings (9%); recordkeeping, reporting, managing family folders (13%); travel between work activities (16%); waiting for clients in the health post (25%); building relationships in the community (13%); and other activities (11%) [54].

### Performance of the health extension program varies across health posts, regions and woredas

The performance of the HEP varies across health posts, regions and woredas. It was found that only 15 (25%) out of 60 health posts were technically efficient. Only 38 (63.3%) health posts exhibited constant returns to scale, and were operating at their most productive scale size. The remaining 22 (36.7%) health posts were considered as scale inefficient [56].

The total time HEWs worked and the proportion of time that they spent on different activities varied significantly among regions (*P* < 0.05). HEWs were, on average, on duty for 15.5 days (73.8%) out of 21 days of observation, and for about 6 h (75%) per day [54].

The performance of the HEP was also variable across woredas. Substantial contrasts were apparent between higher-performing and lower-performing woredas in (1) use of data for problem solving and performance improvement; (2) collaboration and respectful relationships among HEWs, community members, and health center staff; and, (3) coordination between the woreda health office and higher-level regulatory and financing bodies [57].

Performance of the HEWs depended on the support they received from the health systems and the community. HEWs in Adwa, Tigray Region, were more likely to call ambulances than other regions due to support from the HDA and a functioning referral system. In Kafa, Southern Region, HEWs were not utilizing ambulances as they were used for other purposes. In Afar Region, few HEWs were called to assist women as most women gave birth at home with traditional birth attendants [58].

### Social determinants of health affect utilization of health services from the health extension program

In general, the HEP has improved health services coverage and narrowed the inequity among populations. For instance, care seeking at health posts and utilization of iCCM services was unaffected by wealth quintile [35]. More female TB patients were identified in kebeles with trained HEWs than male patients (149.0 vs 91.6, *p* < 0.001). The mean treatment success rate was higher in female patients than in male patients (89.8% vs 81.3%, *p* = 0.05) [20].

On the other hand, a number of factors had statistically significant association with higher utilization of iCCM services at HPs [35]. Childhood immunisation coverage was heterogeneous due to differences in the educational achievement of mothers [13]. Independent predictors of childhood diarrhoea included illiteracy of mothers [OR = 1.74 (95% CI 1.03, 2.91)], monthly family income earn less than 650 Birr [OR = 1.75 (95% CI 1.06, 2.88)], not using soap for hand washing [OR: 7.40 (95% CI 2.61, 20.96)], and being non-MHHs [OR = 4.50 (95% CI 2.52, 8.03)] [24]. The effect of the HEP on regular usage of latrine was maximised when a larger proportion of a village population had attained a primary education [13].

Appropriateness and timeliness of care seeking were influenced by social determinants of health [42]. Women who were literate, listened to the radio, and had income generating activities were more likely to demonstrate good utilization of maternal health services [16]. Women with formal schooling were more likely to use maternal health services than their counterparts [AOR = 5.8, (95% CI 2.1, 16.0)] [34]. Mother’s age, educational status, place of residence, wealth quintile and number of pregnancies were also significantly associated with at least four ANC visits and place of delivery [18]. Mothers from higher income families were more likely to visit health posts [OR = 2.87 (95% CI 1.63, 5.04)] than mothers from lower income families [41].

The HEP has moderately narrowed the inequity, explained by differences in socio-economic status, in health services utilization among populations. However, social determinants of health are still affecting utilization of health services. It is imperative that the program and the health system work with social determinant of health to further improve utilization of services towards universal primary healthcare coverage.

## Discussion

This review and synthesis identified successes and challenges in the implementation and governance of the HEP in Ethiopia. The readiness of the program, the promptness of the systems, and the availability of services are crucial for the success of the HEP. These require health posts, which are well equipped, furnished, and have adequate supplies and functional utilities, and HEWs, who are motivated and capacitated, with improved living and working conditions. In addition, context (including socio-economic status, governance, health system and community), and values and principles of the health system and the community play a significant role for the success of the HEP.

**Fig. 4:**
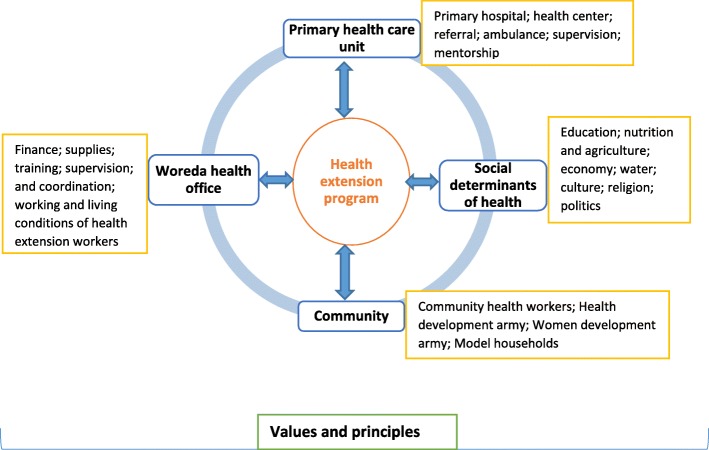
Framework to analyse and improve the performance of the health extension program in Ethiopia

The review also found that the performance of the HEP varies across health posts, regions and woredas. We argue that this variability is an opportunity that the HEP should utilize to systematically assess and analyse the variability and learn lessons toward improved performance of the program and increased coverage of primary healthcare services. We contend that a framework can be developed, based on the evidence from this review and synthesis, to assist the country to improve the performance of its HEP (Fig. 4). The framework emphasises that the success of the HEP depends on activities and performance of: PHCU; woreda health office; sectors of social determinants of health; the political context; and the community. It is vital that all these do their jobs very well and they are coordinated toward an enhanced service delivery, utilization, and improved health (Fig. 4).

The PHCU, including primary hospitals and health centers should be capacitated, linked and coordinated with clear roles and responsibilities to facilitate service delivery, patient referral, supervision and mentorship. This has an implication that investments in one segment of the health system has positive ramifications on the other; and, a weakness of one segment of the health system do also have negative effects on the other. It is thus imperative that the country, regions and woredas capacitate the PHCUs and the health system at large.

The woreda health office should also be capacitated and committed enough to provide finance, supplies, training and supervision to HEWs, coordinate the PHCU and improve the working and living conditions of HEWs. This review has identified that regions and woredas with a strong and well-coordinated PHCU have better implementation of the HEP and improved service utilization at health posts. The country should continue to build its PHCU and implement its agenda for woreda transformation.

The social determinants of health play a vital role in improving the performance of the HEP. They influence the performance of the HEP at two critical points: care seeking, utilization, and health outcomes. This review has identified that regions, woredas and communities with better socio-economic status have better care seeking and utilization and improved health outcomes. It is thus important that the country invests on sectors of social determinants of health toward socio-economic development of its citizens. Moreover, health should be included in the policy, plan and activities of these sectors. The political context in the country has also been vital in the successful implementation of the HEP [59]. Regions with strong political commitment have achieved better results in increasing access to health primary healthcare services than other regions [57, 58].

This review also repeatedly indicates that the community has been playing critical roles in the implementation of the HEP in Ethiopia. The community has become not only a service user but also a service provider in line with the philosophy of the HEP. HEWs supported by strong model households, HDAs, WDAs and other CHWs have been progressively implementing the HEP packages toward universal PHC services. We allude that these initiatives are benefiting the HEP and need to be sustained with improved advocacy and financing.

This review and synthesis has both strengths and limitations. The main strengths are that: (1) it is the first review which assesses the performance of the HEP over time; and, (2) it reviews the four components and 17 packages of the HEP. The main limitations are that: (1) it was not possible to conduct meta-analysis due to absence of adequate studies on the same program area; (2) assessment of risk of bias is not conducted due heterogeneity of the methods used by individual studies; and (2) some studies have a nationwide scale while others have a district scale.

## Conclusion

The community HEP has enabled Ethiopia to achieve significant improvements in maternal and child health, communicable diseases, hygiene and sanitation, knowledge and health care seeking. The success of the HEP hugely depends on readiness and availability of services in health posts which need to be staffed, equipped, furnished, and have adequate supplies and functional utilities. It is also vital that HEWs are motivated, with improved living and working conditions, and capacitated. In addition, socio-economic status, governance, health system and community play a significant role in the successful implementation of the HEP. It is imperative that these lessons are utilized to improve the performance of CHPs towards universal coverage for primary healthcare services.
